# The anti-leprosy drug clofazimine reduces polyQ toxicity through activation of PPARγ

**DOI:** 10.1016/j.ebiom.2024.105124

**Published:** 2024-05-02

**Authors:** Xuexin Li, Ivó Hernandez, Seda Koyuncu, Balázs Kis, Maria Häggblad, Louise Lidemalm, Anna A. Abbas, Sramkó Bendegúz, Anikó Göblös, Lars Brautigam, Jose J. Lucas, Jordi Carreras-Puigvert, Daniela Hühn, Karolina Pircs, David Vilchez, Oscar Fernandez-Capetillo

**Affiliations:** a Science for Life Laboratory, Division of Genome Biology, Department of Medical Biochemistry and Biophysics, Karolinska Institute, S-171 21, Stockholm, Sweden; b Genomic Instability Group, Spanish National Cancer Research Centre (CNIO), Madrid, 28029, Spain; c Cologne Excellence Cluster for Cellular Stress Responses in Aging-Associated Diseases (CECAD), Faculty of Medicine, University of Cologne and University Hospital Cologne, Cologne, Germany; d HCEMM-SU, Neurobiology and Neurodegenerative Diseases Research Group, Budapest, Hungary; e Institute of Translational Medicine, Semmelweis University, Budapest, Hungary; f Centre of Excellence for Interdisciplinary Research, Development and Innovation, University of Szeged, H-6720, Szeged, Hungary; g Zebrafish Core Facility, Karolinska Institute, S-171 21, Stockholm, Sweden; h Center for Molecular Biology, “Severo Ochoa” (CBMSO) CSIC/UAM, Madrid, 28049, Spain; i Networking Research Center on Neurodegenerative Diseases (CIBERNED), Instituto de Salud Carlos III, Madrid, Spain; j Laboratory of Molecular Neurogenetics, Department of Experimental Medical Science, Wallenberg Neuroscience Center and Lund Stem Cell Center, BMC A11, Lund University, Lund, Sweden

**Keywords:** polyQ, Huntington's disease, Chemical screening, PPARγ, Mitochondria

## Abstract

**Background:**

PolyQ diseases are autosomal dominant neurodegenerative disorders caused by the expansion of CAG repeats. While of slow progression, these diseases are ultimately fatal and lack effective therapies.

**Methods:**

A high-throughput chemical screen was conducted to identify drugs that lower the toxicity of a protein containing the first exon of Huntington's disease (HD) protein huntingtin (HTT) harbouring 94 glutamines (Htt-Q_94_). Candidate drugs were tested in a wide range of in vitro and in vivo models of polyQ toxicity.

**Findings:**

The chemical screen identified the anti-leprosy drug clofazimine as a hit, which was subsequently validated in several in vitro models. Computational analyses of transcriptional signatures revealed that the effect of clofazimine was due to the stimulation of mitochondrial biogenesis by peroxisome proliferator-activated receptor gamma (PPARγ). In agreement with this, clofazimine rescued mitochondrial dysfunction triggered by Htt-Q_94_ expression. Importantly, clofazimine also limited polyQ toxicity in developing zebrafish and neuron-specific worm models of polyQ disease.

**Interpretation:**

Our results support the potential of repurposing the antimicrobial drug clofazimine for the treatment of polyQ diseases.

**Funding:**

A full list of funding sources can be found in the acknowledgments section.

**Research in context**

**Evidence before this study**

Despite the general interest in drug repurposing, these efforts have not yet led to efficacious therapies for neurodegenerative diseases. This is also the case for polyQ diseases, for which current options are limited to symptomatic treatments. Regarding the mechanism, previous evidence indicated a role for mitochondrial dysfunction in polyQ disorders and suggested that stimulating mitochondrial biogenesis might serve to alleviate the pathology.

**Added value of this study**

In this study, we reveal that a medically approved antimicrobial, clofazimine, alleviates the toxicity triggered by the expression of polyQ expansions in a wide range of systems, including zebrafish and *C*. *elegans* models of polyQ toxicity. In addition, from the different mechanisms of action that have been previously suggested for clofazimine, our work indicates that its effects in the context of polyQ expansions are related to the activation of mitochondrial biogenesis through PPARγ.

**Implications of all the available evidence**

The evidence presented in this manuscript implies that clofazimine might have therapeutic value in the context of pathologies associated to mitochondrial dysfunction, such as polyQ diseases. Further work in aspects such as improving the delivery of the drug to the central nervous system, or additional experiments in more advanced preclinical models should be conducted to better assess the potential value of this therapeutic strategy.

## Introduction

Polyglutamine (polyQ) diseases include 9 inherited hereditary neurodegenerative syndromes that are caused by the expansion of Q-coding repeats within the exons of several seemingly unrelated genes.^1^ One of these pathologies is Huntington's disease (HD), a rare neurodegenerative disease with an incidence of 3–5 cases per 100,000 people worldwide.^2^ In HD, the disease is linked to the expansion of a CAG repeat within the first exon of huntingtin (*HTT*), which becomes pathogenic above 35 repeats, with the severity of the disease correlating with repeat length.^3^^,^^4^ Although HTT dysfunction has been proposed to contribute to HD,^5^^,^^6^ an alternative hypothesis is that the pathology is caused by gain-of-function toxicity of polyQ-bearing mutant HTT (mHTT). In support of this view, early studies showed that transgenic mice expressing a fragment of exon 1 of mHTT, including the expanded polyQ track, suffer from motor dysfunction and premature death.^7^^,^^8^ Furthermore, seminal work revealed that ectopic expression of polyQ expansions inserted in *HPRT*, a gene not mutated in patients, also leads to neurodegeneration and premature death, highlighting the causal role of polyQ toxicity regardless of *HTT*.^9^

In what regards to the mechanisms driving polyQ toxicity, this remains to be fully understood. An important feature of these expansions is their propensity to form insoluble aggregates that form intraneuronal inclusions, which are found in mouse models and patients from several polyQ diseases including HD.^10^, ^11^, ^12^ However, whether these inclusions are the real cause of the pathology has been the subject of intense debate, and it is evident that mHTT can also be toxic independent of the formation of large aggregates (reviewed in^1^). Regardless of the inclusions, mHTT has been shown to drive multiple cellular alterations, affecting mRNA transcription,^13^, ^14^, ^15^ protein degradation and post-translational modifications,^16^ as well as synaptic function, plasticity^17^, ^18^, ^19^, ^20^, ^21^ and mitochondrial activity.^22^, ^23^, ^24^, ^25^, ^26^

Unfortunately, these mechanistic discoveries have not led to clinical improvements in HD treatment. The only approved treatments for HD, tetrabenazine and deutetrabenazine, alleviate involuntary movements (chorea) but do not cure the disease.^27^^,^^28^ Therefore, there is an urgent need to identify therapies for polyQ diseases, which is an area of intense research. Efforts are spread among strategies aimed at preventing mHTT aggregates or promoting their clearance as well as targeting their downstream pathological effects (reviewed in^29^). Notably, several unbiased chemical screens have focused on the identification of compounds that lower polyQ aggregates in biochemical assays, which often leads to compounds that show toxicity when subsequently evaluated in *in vivo* models.^30^^,^^31^ Here, we present the results of a high-throughput imaging-based drug-repurposing screening aimed at identifying compounds that reduce the toxicity of polyQ expansions.

## Methods

### Cell culture and reagents

U2OS (human osteosarcoma) cells (RRID:CVCL_0042) were obtained from ATCC and cultured in DMEM + Glutamax (Thermo Fisher Scientific), 10% FBS and 1% Penicillin/Streptomycin. U2OS^Q94^ cells were cultured in DMEM + Glutamax supplemented with 10% Tet system approved FBS (Takara, 631,368) and 1% penicillin/streptomycin, selected with zeocin and Blasticidin S. KBM7 cells (RRID:CVCL_A426) were a kind gift from Thijn Brummelkamp (The Netherlands Cancer Institute). KBM7^Q94^ and KBM7-mCherry cells were cultured in IMDM (Thermo Fisher Scientific), supplemented with 10% FCS and 1% penicillin/streptomycin. SH-SY5Y human neuroblastoma cells (RRID:CVCL_0019) were obtained from ATCC. SH-SY5Y^Q94^ cells were cultured in DMEM/F-12 (Thermo Fisher Scientific), 10% Tet system approved FBS and 1% Penicillin/Streptomycin. For the transfection of U2OS cells, Lipofectamine2000 transfection reagent (Thermo Fisher Scientific) was used, following the standard protocol. For the transfection of KBM7 cells, the Amaxa Nucleofector kit (Reactive L, X-001 program) was used, and 5 × 10^5^ cells with 10 μg of the indicated plasmids were transfected according to the manufacturer's protocol. For lentiviral transduction of SH-SY5Y cells, the pLVX-UbC-rtTA-Htt-Q94-CFP vector was co-transfected into HEK293T cells (RRID:CVCL_0063; ATCC) using Lipofectamine2000 with packaging vector pMD2.G (Addgene, #12259) and psPAX2 (Addgene, #12260). Lentiviral supernatants were collected 36 h after transfection, filtered, and used immediately for transduction. All cell lines were subjected to STR profiling and grown at 37 °C in a humidified atmosphere with 5% CO_2_.

Adult dermal fibroblasts from 7 control donors (3 males age 44, 61 and 71, and 4 females 29, 34, 39 at biopsy) were obtained from the Huntington's disease clinic at the John van Geest Centre for Brain Repair (Cambridge, UK) and from HCEMM-Szeged University Skin Research Group, (Hungary, Biobank ID: 001335998) and used under ethical approvals REC 09/H0311/88 and IV/2625–1/2021/EKU. Adult dermal fibroblasts were kept in DMEM + Glutamax medium (Gibco) supplemented with 10% FBS (Gibco) and 1% penicillin/streptomycin (Gibco) as previously described.^32^

### Plasmids

The human Htt-exon1-Q94 fragment from pTreTight-Htt94Q-CFP (Addgene, #23966) was cloned into pBlueScript SK+ (a kind gift from Eva Brinkman) using the HindIII and BamHI sites to generate an intermediate plasmid, pBlueScript SK-polyQ94. PINTO-polyQ94-GFP was cloned using pBlueScript SK-polyQ94 and pINTO-N-GFP using the KpnI and NotI sites. pcDNA3.1-EGFP-poly94 was cloned using pcDNA3.1-mCherry (Addgene, #128744)^33^ and pINTO-polyQ94-GFP with AflII and NotI sites. To clone the plasmid for SH-SY5Y cell infection, polyQ94-CFP was cloned into pBlueScript SK + using the XbaI and EcoRI sites to generate the intermediate plasmid SK-polyQ94-CFP. pLVX-UbC-rtTA-polyQ94-CFP was cloned by SK-polyQ94-CFP and pLVX-UbC-rtTA-Ngn2:2A:Ascl1 (Addgene, #127289) using the dual NotI sites. The genetic construct of the zebrafish plasmid was based on the vector pDEST-Tol2-PA2-CMV-AB-mCh (Addgene, #160435),^34^ in which the Aβ peptide was exchanged with EGFP-polyQ94 from pcDNA3.1-EGFP-polyQ94. The lentiviral packaging vector, pMD2.G (Addgene, #12259) and psPAX2 (Addgene, #12260) were used.

### High-throughput screening (HTS)

Plate and liquid handling was performed using Echo550 (Labcyte, USA), Viaflo 384 (Integra Biosciences, Switzerland), Multiflo FX Multi-Mode Dispenser (BioTek, USA), and a Hydrospeed washer (Tecan, Switzerland). Cells were seeded in black 384-well plates with clear bottom (BD Falcon, #353962). Compound libraries were provided by the Chemical Biology Consortium Sweden (CBCS). The chemical collection used in the primary screening contained 1122 medically approved compounds from the Prestwick library and 94 epigenetic drugs available at CBCS collections (the list of compounds is available in Table S1). For the primary screening, U2OS^Q94^ cells were trypsinized and resuspended in culture medium. The cell suspension (100 cells in 30 μl/well) was dispensed into 384-well plates and exposed to a final concentration of 1 μM of compounds diluted in dimethyl sulfoxide (DMSO) for 8 days. Cells were fixed with 4% PFA and nuclei were stained with 2 μM Hoechst 33342 for 15 min in the dark.

Plates were imaged using an IN Cell Analyzer 2200 system (GE Healthcare, USA) with a 10 × objective, and four images per well were acquired, covering the entire well. Images were analysed with the open-source software CellProfiler^35^ using a custom-made pipeline for the detection of nuclei count. All values were normalized to the DMSO conditions within each plate. The mean value for each compound in triplicate was calculated, representing a single measurement per compound. For the validation screen, U2OS^Q94^ cells were exposed to four concentrations (0.5, 1, 3, and 10 μM) of the selected hits for eight days. The validation was conducted in triplicate, and the images were analysed as described above. Statistical analysis of imaging data was conducted using Graphpad Prism software.

### Immunoblotting

Cell pellets were lysed in RIPA buffer (Thermo Fisher Scientific) supplemented with protease and phosphatase inhibitor cocktail (Roche), sonicated for 5 min, and centrifuged at 14000 rpm at 4 °C for 15 min. 30 μg whole-cell extracts were separated by SDS–PAGE and transferred onto a nitrocellulose membrane (Bio-Rad). After blocking in 5% milk in TBS-T, indicated antibodies were diluted in blocking buffer and incubated overnight at 4 °C. The following dilutions of primary antibodies were used: GFP (1:300, Abcam, #ab290, RRID:AB_303395), Polyglutamine (1:1000, Sigma–Aldrich, #P1874, RRID:AB_532270), PPAR-γ (1:250, Abcam, #ab45036, RRID:AB_1603934), Vinculin (1:2000, Abcam, #ab130007, RRID:AB_11156698). The signal associated to HRP-conjugated secondary antibodies (ThermoFisher, mouse #31430 (RRID:AB_228307) and rabbit #31460 (RRID:AB_228341)) was developed with a SuperSignal West Pico PLUS Chemiluminescent Substrate kit (ThermoFisher, #34580), and analysed in an Amersham Imager 600.

### Immunofluorescence

For MitoTracker stainings in U2OS^Q94^ cells, medium was removed from the dish followed by the addition of prewarmed (37 °C) staining solution containing MitoTracker probe (100 nM, M7512, ThermoFisher) for 30 min. Cells were fixed with 4% PFA for 15 min and subsequently stained with Hoechst 33342 (Sigma, 14533), followed by image acquisition. For SH-SY5YQ94 cells MitoTracker staining, medium was removed from the dish, and prewarmed (37 °C) staining solution containing 300 nM MitoTracker was added. After 30 min incubation, cells were fixed with 4% PFA for 15 min and permeabilized with 0.1% Triton X-100 for 10 min at room temperature, subsequently stained with Hoechst 33,342 (Sigma, 14,533). After blocking (3% BSA and 0.1% Tween-20 in PBS), the indicated antibodies were applied, TUBB3 (1:1000, Biolegend, 801202, RRID:AB_2728521). After staining with Hoechst 33,342 (Sigma, 14,533), images were acquired and analysed.

Immunocytochemistry to stain iNs was performed as previously described.^32^ Mitochondria was stained using MitoTracker™ Deep Red FM (ThermoFisher, M22426) at a dilution of 1:1000. iNs were incubated in MitoTracker solution diluted in LCM for 15 min in the dark. Cells were washed with phosphate-buffered saline (PBS) once and fixed by 4% paraformaldehyde solution for 10 min. Permeabilization was done using 0.1% Triton X-100 in PBS for 10 min. Blocking was done using donkey serum in PBS (50 μl/1 ml) for 30 min. Anti-rabbit TAU (Genetex, GTX130462, RRID:AB_2886280) and Anti-chicken MAP2 (Abcam, #ab5392, RRID:AB_2138153) primary antibodies were used to stain mature neurons. The plates were incubated overnight at 4 °C in 1:500 (TAU) and 1:5000 MAP2 antibody-serum solution. The following day cells were washed twice with PBS and stained using Alexa Fluor® 488 (AffiniPure Donkey Anti-Rabbit IgG) (Jackson ImmunoResearch, RRID:AB_2492289) and Cy™3 (AffiniPure Donkey Anti-Chicken IgY (IgG) (H + L)) (Jackson ImmunoResearch, RRID:AB_2340363) diluted in blocking solution at 1:200 for 2 h in dark. Following this DAPI staining was done for 15 min (1:1000) and washed with PBS. Finally, high-content automated microscopy analysis was performed using Thermo Scientific Cell Insight CX5 system.

Image analysis of iNs was done using the Thermo Scientific CellInsight CX5 system to determine DAPI^+^, TAU^+^ cell number, purity, and conversion efficiency by “neuronal profiling” (NP). NP was done using 20× objective. DAPI^+^ cells were identified based on intensity, area, and shape. TAU^+^ cells were identified based on intensity, area, neuronal morphology on a cell-by-cell basis. Validation was done by excluding border objects and cells with abnormal area, shape and intensity. Purity was given by the fraction of TAU^+^ to DAPI^+^ cells. Efficiency was given by the fraction of TAU^+^ cells to the number of plated fibroblasts. To determine average mitochondrial “spot” intensity neuronal profiling was used on images acquired by 20× objective. Valid nuclei were determined by DAPI staining, valid neurons were determined using TAU. Spots were validated by excluding border objects and cells with abnormal area, shape and intensity. Mitochondria “spots” were only analysed in valid neuronal cell bodies and neurites.

### Mitochondrial membrane potential assessment

U2OS^Q94^ cells were treated as indicated and seeded into a 96-well imaging plate (Agilent). Next day, growth medium (without FCS) containing 2 μM JC-1 probe (Thermo Fisher Scientific) was added and incubated for 30 min in the dark at 37 °C, 5% CO2 and 5% O2 atmosphere. Cells were washed twice with PBS and imaged immediately using an InCell 2200 microscope. Signal intensity of JC-1 emission at 590 nm was analysed with CellProfiler software.^35^

### Transmission electron microscopy

For the TEM analyses, U2OSQ94 cells were plated on 15 cm petri dishes. The following day, cells were fixed at RT in 2% glutaraldehyde in 0.1 M phosphate buffer, pH 7.4. After fixation, the cells were rinsed in 0.1 M phosphate buffer and centrifuged. Cell pellets were post-fixed in 2% osmium tetroxide in 0.1 M phosphate buffer, pH 7.4 at 4 °C for 2 h. Cells were then stepwise dehydrated in ethanol, followed by acetone and finally embedded in LX-112. Ultrathin sections (∼50–60 nm) were prepared using a Leica EM UC7 and contrasted with uranyl acetate followed by lead citrate. TEM imaging was done in a Tecnai 12 Spirit Bio TWIN transmission electron microscope operated at 80 kV and digital images acquired using a Veleta camera (Olympus Soft Imaging Solutions).

### Flow cytometry

For the analysis of the competition assay of KBM7^Q94^ and KBM7-mCherry in, 4 × 10^4^ KBM7^Q94^ and KBM7-mCherry cells were mixed at a 1:1 ratio and were seeded in T175 flasks. Cells were treated with CFZ and TZD at indicated concentrations. After 5 or 15 days, cells were analysed for green or red fluorescence by flow cytometry (Bio-Rad S3e cell sorter). Data were processed with the Flow Jo 10 software to calculate the percentage of each cell population.

### Viability assays

For clonogenic survival assays, 500 cells per well were plated in 6-well tissue culture plates in the corresponding culture medium. Cells were treated with the indicated concentrations of drugs and maintained with the compounds for 12 days, changing the medium every 3–4 days, and then fixed and stained with 0.4% methylene blue in methanol for 30 min.

### RNA-seq and data analysis

Total RNA was extracted from cell pellets using a Purelink RNA Mini Kit (Invitrogen #12183025) following manufacturer's instructions. Total RNA was subjected to quality control with Agilent Tapestation (#G2991BA). To construct libraries suitable for Illumina sequencing, an Illumina stranded mRNA prep ligation sample preparation protocol was used with a starting concentration of total RNA between 25 and 1000 ng. The protocol includes mRNA isolation, cDNA synthesis, ligation of adapters and amplification of indexed libraries. The yield and quality of the amplified libraries was analysed using a Qubit by Thermo Fisher and the quality of the library was checked using the Agilent Tapestation. Indexed cDNA libraries were normalized and combined, and pools were sequenced using an Illumina platform. STAR^36^ was used for sequence alignment based on the GRCh38 DNA primary assembly reference build,^37^ and quantification was done using feature Counts^38^ with reference build GRCh38.101.^37^

Differential expression (DE) analyses between the groups were performed using DESeq2.^39^ Generalized linear model (GLM) was fitted to the expression data and shrunken log2fold-change (LFC) using adaptive Student's t prior shrinkage estimator.^39^^,^^40^ Multiple testing correction was done using Benjamini-Hochberg (BH) method.^41^ GSEA analysis was performed on the gene-level statistics from the DE analyses results against the molecular signatures from the Molecular Signatures Database (MSigDB) v7.5.1^42^^,^^43^ and the Reactome database.^44^ Specifically, signatures from the ontology gene set C5 of MSigDB, containing Gene Ontology (GO)-derived gene sets,^45^^,^^46^ as well as the complete gene sets from the Reactome database, retrieved from fgsea 1.20.0 (retrieved from the preprint of^47^), were used. GSEA analysis was carried out using clusterProfiler 4.2.2^48^ to identify enriched terms. The transcriptional signatures, identified to be the sets of top up/down-regulated 150 genes (BH p-adjusted values < 0.05, ranked by LFC) from the DE analysis outcomes, were separately used as inputs to the Connectivity Map (CMap) Query clue.io tool (https://clue.io/)^49^ to identify drugs with signatures similar to that clofazimine.

### Molecular docking

The 3D crystal structure of PPAR-γ was downloaded from the Protein Data Bank (http://www.rcsb.com.#3ET0). Autodock vina was used to remove water molecules and add missing side or back chains and residues. Chemical structures were downloaded from the ZINC database of molecular structures for virtual screens (www.zinc15.org). Molecular docking was conducted by Auto Dock vina and the binding energy of each ligand was analysed based on binding free energies and root mean square deviation (RMSD) values. Top nine binding energies of each ligand were listed.

### Direct reprogramming

Induced Neurons were produced by using an all-in one lentiviral vector LV.U6.shREST1.U6.shREST2.hPGK.BRN2.hPGK.Ascl1.WPRE. The cells were plated onto 96-well plates (Nunc Delta surface treated plates (Thermo Scientific)) at 8000 cells/well density in fibroblast medium and one day later (Day 0) transduced at MOI 20 using the all-in-one lentiviral vector. On day 3 the fibroblast medium was changed to Early Conversion Medium (ECM): nDIFF (NDIFF 227) (Takara) supplemented with 1% penicillin/streptomycin (Gibco) and a cocktail of small molecules and growth factors as previously described.^32^ Every second day 15–20% of the medium was changed to fresh ECM until day 17. On day 17 full media change was done to Late Conversion Medium (LCM) which contains small molecules. The same protocol of medium change was followed until day 27.

### Zebrafish study

To test the impact of CFZ in alleviating polyQ toxicity in zebrafish. On day 0, the injection mix (25 ng/μl transposase, 50 ng/μl vector, 0.3% phenol red) was injected into 150 eggs. The compounds were added in to the E3 medium at 12.5 μM, CFZ treatment naïve injected group and DMSO treated un-injected group were also included. After 24 h, dead embryos/well were counted and imaged. The experiment was triplicated.

### *C. elegans* bending assay

AM716 (*rmIs284[pF25B3.3::Q67::YFP]*) strain was generously gifted by R.I. Morimoto. These worms were grown and maintained at 20 °C using standard methods.^50^ To synchronize the strain, the bleaching technique was used.^51^ Following this process, the eggs were kept on M9 buffer without food overnight to allow hatching but prevent further development. Synchronized L1 larvae were collected and randomly transferred onto plates with OP50 bacteria covered with a final concentration of 5 μM CFZ or vehicle control (DMSO) at 20 °C until late day 1 of adulthood. After 5 μM CGZ or vehicle control (DMSO) treatment, day 1 adult worms were picked and transferred to a drop of M9 buffer. After 30 s of adaptation, the number of body bends was counted for the next 30 s. A body bend was defined as a change in direction of the bend at the mid-body.^52^^,^^53^

### Ethics

Work with the human adult dermal fibroblasts from control donors, who had signed an informed consent, was done under ethical approvals REC 09/H0311/88 and IV/2625-1/2021/EKU. For zebrafish work, all animals were housed and used for experiments in compliance with EU and Swedish regulations, and the breeding stock was maintained under the ethical license #15591/2023 issued by the Stockholm ethical council.

### Statistics

Statistical parameters and tests are reported in the Figures and corresponding Figure Legends. Statistical analysis was done using GraphPad Prism version 8.0 (GraphPad Software Inc). One-way-ANOVA was performed for all the datasets that required comparison among multiple data points within a given experimental condition.

### Data availability

RNA sequencing data associated to this work are accessible at the GEO repository, under accession number GSE222758.

### Role of funders

The funders played no roles in the study design, data collection, data analysis, interpretation, or the writing of the manuscript.

## Results

### A chemical screen for modulators of polyQ-toxicity

**Fig. 1:**
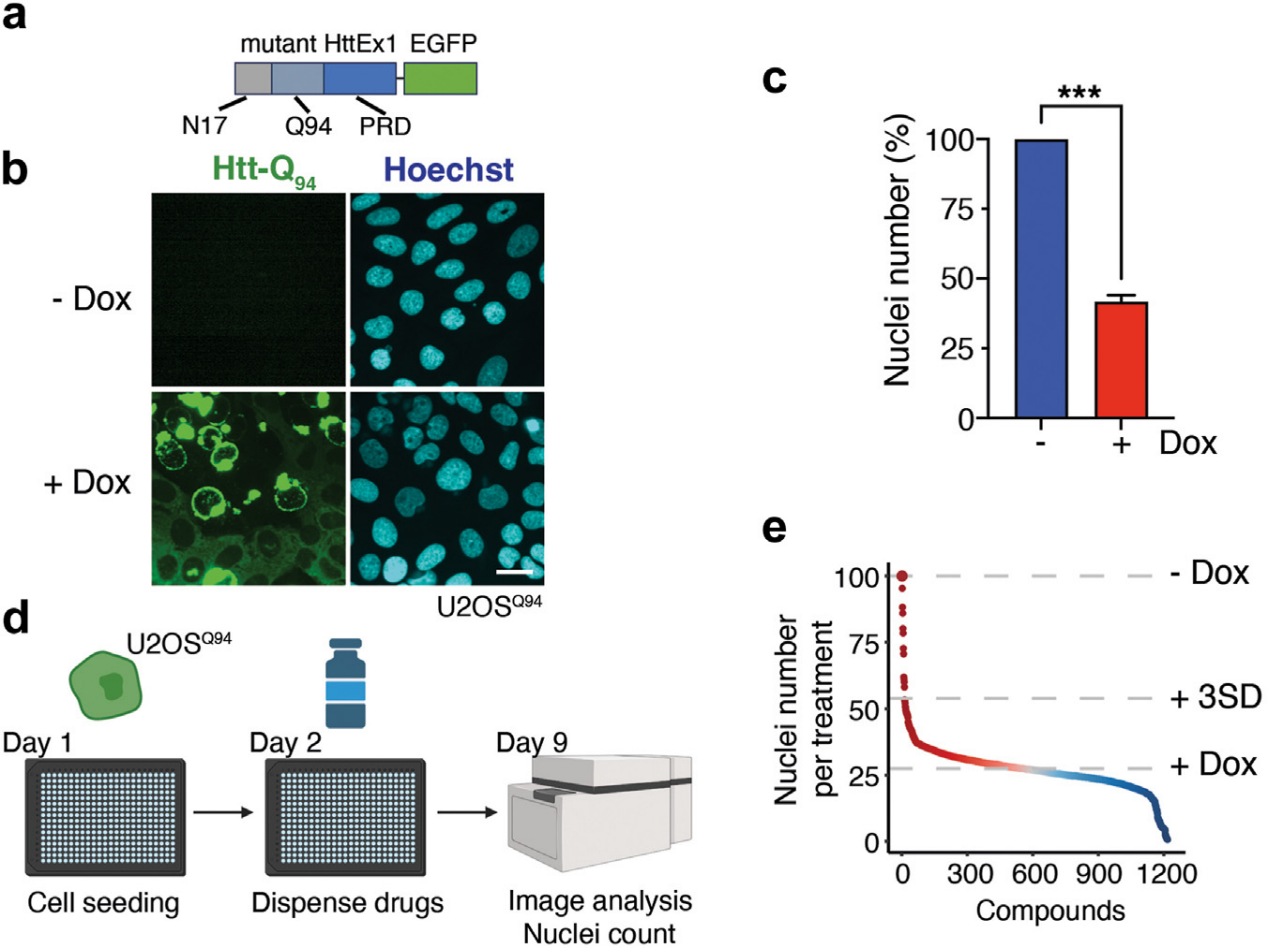
**A chemical screen to identify modifiers of polyQ toxicity.** (**a**) Scheme of the Htt-Q_94_ fusion protein expressed in U2OS^Q94^ cells, which contains the first exon of human HTT, including the first 17 amino acids (N17), a stretch of 94 glutamines (Q94) and the proline-rich domain (PRD), fused to EGFP. (**b**) Representative image of Htt-Q_94_ expression (monitored by EGFP, green) in U2OS^Q94^ cells treated or not with dox (50 ng/ml) for 8 days. Hoechst (blue) was used to stain DNA and enable the quantification of nuclei. (**c**) High-Throughput Microscopy (HTM)-mediated quantification of nuclei numbers from the data presented in (**a**). The screening was only done once (n = 1), and at least 5000 nuclei were counter per condition. Error bars indicate SD. ∗∗∗*p* < 0.001, t-test. (**d**) Pipeline of the chemical screen. On day 0, U2OS^Q94^ cells were seeded on 384-well plates. On the following day, cells were treated with dox (50 ng/ml) and with the compounds from the library at 1 μM. Nuclei numbers were quantified by HTM on day 9. Scattered controls of cells not treated with dox, or only treated with dox but without additional compounds were used for normalization. (**e**) Hit distribution of the screen described in (**d**). Compounds that led to an increase in nuclei numbers higher than 3SD when compared to the numbers founds on wells only treated with dox were taken for secondary validation (Fig. S1).

To conduct a chemical screen, we first generated an inducible system enabling the expression of an EGFP fusion protein containing the first exon of human HTT with an expanded polyQ tract of 94 glutamines (Htt-Q_94_ hereafter) (Fig. 1a). The cDNA was cloned in a Tet-On gene expression system to enable the expression of Htt-Q_94_ upon addition of doxycycline (dox). Since polyQ expression is toxic not only in neurons but also in any cell type, the system was stably integrated into human osteosarcoma U2OS cells, which are widely used in large chemical screens, and a clone with stringent regulated expression was selected for further experiments (U2OS^Q94^). Before conducting the screening, we confirmed dox-inducible expression of Htt-Q_94_, as evidenced by the widespread accumulation of EGFP-expressing cells (Fig. 1b). Moreover, as previously reported in similar setups, a 1-week treatment with dox led to the appearance of cells with Htt-Q_94_ aggregates. At this time, Htt-Q_94_ expression led to a significant reduction in cell numbers, as quantified by nuclear detection using High-Throughput Microscopy (Fig. 1c), thereby confirming the toxicity of polyQ expression in this cell system.

The chemical library integrated 1200 FDA-approved compounds and 94 additional drugs targeting components of the epigenetic machinery (Table S1). We added epigenetic drugs to our screening set because several of these drugs have been found to have potential for the treatment of HD and other neurodegenerative diseases.^54^ To conduct the screening, U2OS^Q94^ cells were seeded in 384 well plates at 100 cells/well and treated with dox (50 ng/ml) and the library compounds (1 μM) for 8 days. At this point, cells were fixed and DNA was stained with Hoechst, enabling the quantification of nuclei (Fig. 1d and e). 35 compounds leading to an increase in nuclei numbers larger than three standard deviations (SD) from those found in the control wells (only treated with dox) were selected for a subsequent dose–response validation screen, which was conducted at 0.5, 1, 5, and 10 μM. From the secondary screen, four compounds showed significant rescue of Htt-Q_94_ toxicity in at least two of the doses tested: promethazine (PRM), amodiaquine (AMD), clofazimine (CFZ), and troglitazone (TZD) (Fig. S1a).

### Clofazimine and troglitazone rescue polyQ-toxicity in vitro

**Fig. 2:**
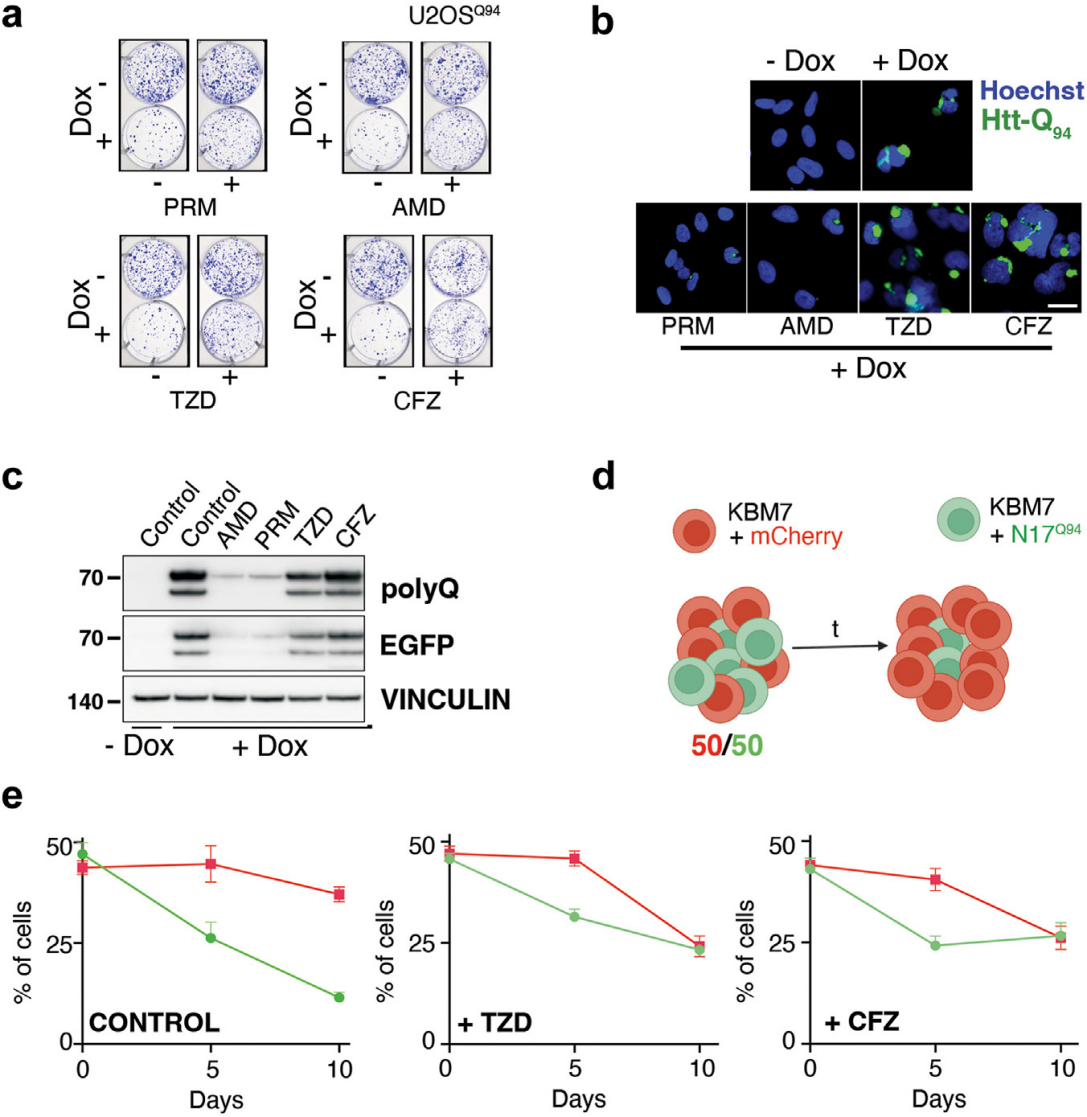
**Clofazimine and troglitazone alleviate polyQ toxicity in vitro.** (**a**) Representative images from clonogenic survival assays performed in U2OS^Q94^ cells, treated or not with dox (50 ng/ml) and the indicated drugs at 2 μM for 12 days. The full dose–response dataset from the clonogenic assays is available at (Fig. S2). (**b**) Representative images of Htt-Q_94_ expression (detected by the EGFP signal, green) in U2OS^Q94^ cells, treated or not with dox (50 ng/ml) and the indicated drugs at 2 μM for 8 days. Nuclei were stained with Hoechst (blue). Scale bar (white), represents 2 μm. (**c**) WB analysis of Htt-Q_94_ expression levels, monitored both with an anti-EGFP antibody or an antibody against polyQ peptides, in the experiment defined in (**b**). Vinculin levels were assessed as a loading control. (**d**) Scheme of the competition assay using KBM7 cells expressing either mCherry (red, control) or a fusion protein between Htt-Q_94_ and EGFP (green). When co-cultured, the percentage of Htt-Q_94_ progressively declines. (**e**) Data from the KBM7 competition experiment defined in (**d**), in cultures treated with DMSO (control), TZD or CFZ (at 5 μM). Error bars indicate SD (n = 3).

Next, to evaluate whether the compounds were able to exert a prolonged effect in reducing the toxicity associated with Htt-Q_94_ expression, we conducted clonogenic survival assays. These experiments confirmed that all four compounds increased the number of colonies in dox-treated U2OS^Q94^ cells (Fig. 2a and Fig. S2a). Before entering into mechanistic analyses, we first wanted to discard hits that were acting by preventing dox-dependent expression of Htt-Q_94_, an issue that we previously faced when conducting chemical screens based on Tet-On expression.^55^ To this end, we first measured their effects on dox-induced gene expression in an independent Tet-On system that enables the expression of a TDP43-GFP fusion. In contrast to loperamide, which inhibits dox-dependent gene expression,^55^ none of the four hits affected TDP43-EGFP two days after exposure to dox (Fig. S2b). However, the formation of aggregates in U2OS^Q94^ cells takes around 1 week of dox exposure, and at this time PRM and AMD significantly limited Htt-Q_94_ levels, as measured by immunofluorescence (IF) and western blotting (WB), whereas CFZ and TZD did not affect Htt-Q_94_ expression (Fig. 2b and c). Even if these results suggest that AMD and PRM might promote the clearance of polyQ aggregates, for this study we focused on TZD and CFZ to understand how these compounds limit polyQ toxicity despite not affecting its levels.

To further validate these results in vitro using an orthogonal model, we performed growth competition assays in the human leukemic KBM7 cell line. To this end, we co-cultured KBM7 cells expressing either mCherry or EGFP-Htt-Q_94_ (KBM7^Q94^) for 10 d. In the absence of drugs, the percentage of KBM7^Q94^ cells progressively declined, confirming that Htt-Q_94_ expression also impairs cellular fitness in this model. In contrast, treatment with CFZ or TZD rescued the relative decline of KBM7^Q94^ cells, confirming the in vitro effects of both drugs in alleviating polyQ toxicity (Fig. 2d and e). Interestingly, one of these two compounds, TZD, is a well-established agonist of the peroxisome proliferator activated receptor gamma (PPARγ), a target that has been previously studied as a potential therapy for various neurodegenerative diseases including HD, confirming the usefulness of our screen to identify potential therapies.^26^^,^^56^, ^57^, ^58^, ^59^ CFZ is an antibiotic originally developed as a treatment for leprosy that is active against a wide range of mycobacteria^60^; however, this drug has not been previously investigated in the context of neurodegeneration. Thus, we selected CFZ for further experiments.

### Clofazimine rescues polyQ-induced mitochondrial damage

**Fig. 3:**
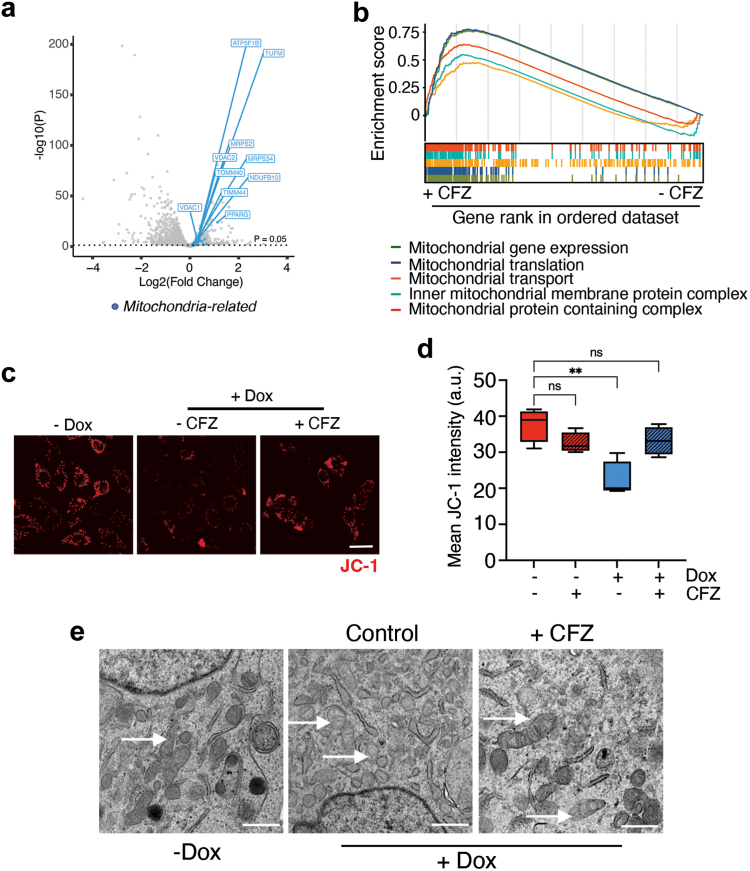
**Clofazimine restores mitochondrial function in polyQ-expressing cells.** (**a**) Volcano plot representing RNA-seq data illustrating the impact of CFZ treatment (5 μM; 8 days) in dox-induced U2OS^Q94^ cells. Genes above dotted line are differentially regulated (*p* < 0.05). Blue dots highlight mitochondria-related genes. (**b**) GSEA analyses from the experiment defined in A, illustrating the overall increase in mitochondria-related pathways upon CFZ treatment in dox-induced U2OS^Q94^ cells. (**c**) Representative images the JC-1 signal (red) in U2OS^Q94^ cells treated or not with dox (50 ng/ml) and CFZ (5 μM). Nuclei were stained with Hoechst (blue). Scale bar (white), represents 5 μm. (**d**) HTM-dependent quantification of mean JC-1 signal per cell from the experiment defined in (**c**). Error bars indicate SD and black lines indicate media values (n = 3). ∗∗*p* < 0.01, n.s., non-significant, t-test. (**e**) Representative images from transmission electron microscopy of U2OS^Q94^ cells treated or not with dox (50 ng/ml) and CFZ (5 μM). Arrows indicate mitochondria, which are significantly altered upon expression, and improved upon a concomitant treatment with CFZ. Scale bar (white), represents 0, 5 μm.

To understand how CFZ treatment rescued polyQ toxicity, we conducted transcriptomic analyses by RNA sequencing (RNA-seq) in dox-induced U2OS^Q94^ cells treated with or without CFZ for 8 days. Interestingly, these analyses revealed a general impact of CFZ in boosting the expression of multiple factors related to mitochondria, such as voltage-dependent ion channels, translocases, subunits of ATP synthase, and components of mitochondrial translation (Fig. 3a). Consistently, Gene Set Enrichment Analyses revealed that CFZ treatment led to significant enrichment of multiple pathways related to mitochondrial function (Fig. 3b) in dox-treated U2OS^Q94^ cells.

To evaluate mitochondrial activity, we used JC-1, a ratiometric dye that enables the monitoring of mitochondrial membrane potential.^61^ Using this approach, we observed that Htt-Q_94_ expression reduced mitochondrial membrane potential in dox-treated U2OS^Q94^ cells in a manner that was significantly rescued by CFZ (Fig. 3c and d). Similar results were obtained with Mitotracker, an independent dye that makes covalent bonds and can thus be used to stain mitochondria,^62^ suggesting that Htt-Q_94_ expression might in fact reduce total mitochondrial mass (Fig. S3). Consistently, transmission electron microscopy analyses revealed that Htt-Q_94_ expression had a profound impact on the mitochondria of U2OS^Q94^ cells, characterized by swelling and substantial abnormalities in the external membranes and cristae, all of which were rescued by CFZ (Fig. 3e).

### The effect of clofazimine is mediated by PPARγ activation

**Fig. 4:**
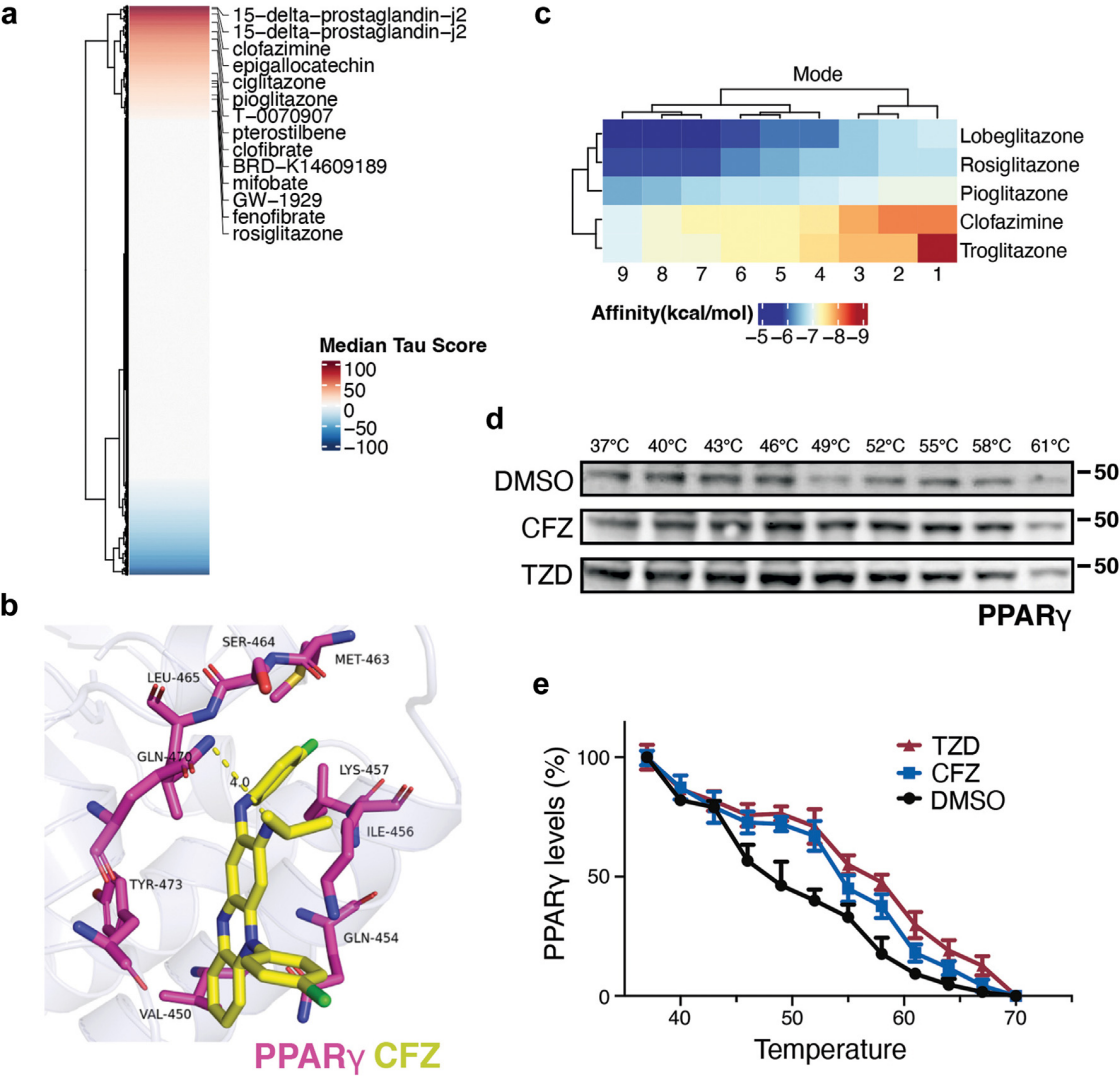
**Clofazimine activates PPARγ.** (**a**) The transcriptional signature of CFZ-treated cells was used as input to search for drugs exerting a similar transcriptional signature at the Connectivity Map database from the Broad Institute at MIT.^49^ The panel indicates an enrichment of PPARγ agonists among the drugs showing a transcriptional signature resembling that of CFZ. (**b**) Molecular docking illustrating the fitting of CFZ (yellow) in an allosteric pocket of PPARγ (red). The interaction occurs through hydrophobic forces and the formation of a hydrogen bond with gln-470 (length of the bond, 4.0 Å). CFZ has hydrophobic interactions with tyr-473, val-450, gln-454, ile-456, lys-457, met-463, ser-464 and leu-465. (**c**) Binding affinities of CFZ and several PPARγ agonists towards PPARγ, based on the molecular docking experiment shown in (**b**). (**d**) Cellular thermal shift assay (CETSA) measuring the effects of TZD and CFZ on PPARγ levels at increasing temperatures. Both compounds increased the thermal stability of PPARγ when compared to the DMSO control. (**e**) Quantification of the CETSA studies shown in (**d**). Error bars indicate SD.

CFZ has been used as an antimycobacterial since the 1950s.^63^ In addition, recent screens have also identified that CFZ prevented infection by a wide range of viruses, including SARS-CoV-2.^64^ Regarding its mechanism of action, several possibilities have been proposed such as the generation of oxidative stress or the inhibition of bacterial DNA replication by binding to guanine bases. In addition, a recent study revealed that CFZ could bind to and activate PPARγ, a major regulator of mitochondrial biogenesis.^65^ To investigate which of these different effects, if any, could be related to the rescue of polyQ toxicity, we used the transcriptional signature of CFZ-treated U2OS cells to interrogate the Connectivity Map (CMap), a database from the Broad Institute at MIT that stores the transcriptional signatures of more than 5000 drugs.^49^ These analyses revealed an enrichment of PPARγ agonists among the compounds presenting a transcriptional signature similar to that of CFZ (Fig. 4a). In fact, PPARγ itself was transcriptionally induced by CFZ in our transcriptomic analyses (Fig. 3a). In agreement with our bioinformatic analyses, molecular docking revealed that CFZ is able to bind to the same pocket in PPARγ as other agonists, such as thiazolidinediones, with a similar binding affinity (Fig. 4b and c). Furthermore, cellular thermal shift assays (CETSA)^66^ indicated that TZD and CFZ have a similar impact in stabilizing PPARγ at increasing temperatures, supporting in vivo target engagement (Fig. 4d and e). Together, these results indicated that the effect of CFZ in rescuing polyQ toxicity is related to PPARγ activation.

### In vitro effect of clofazimine in neurons

**Fig. 5:**
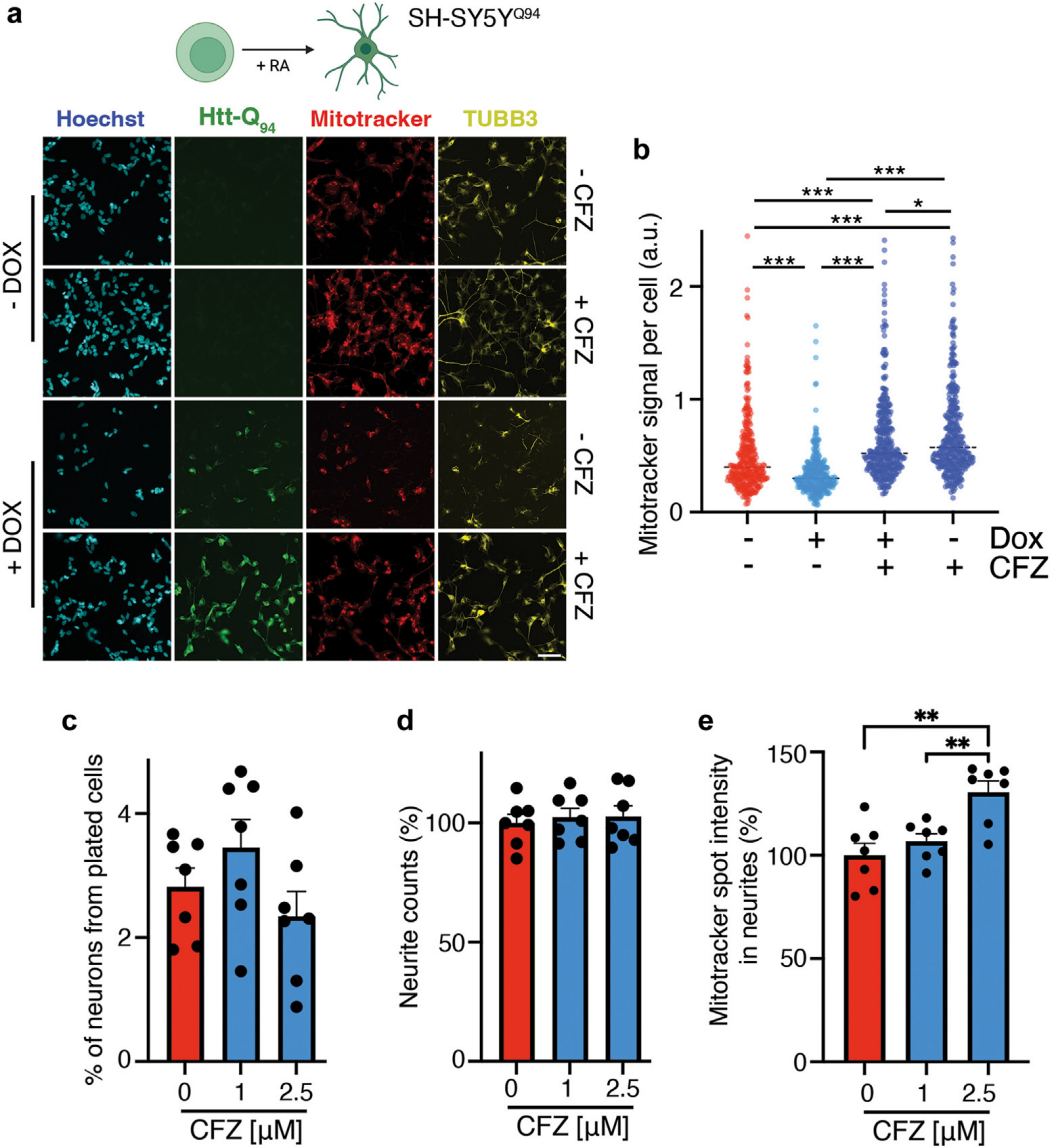
**Effect of CFZ in neurons in vitro.** (**a**) Representative images of SH-SY5Y^Q94^ cells differentiated with RA (10 μM, 5 days), and subsequently treated with dox (35 ng/ml) with or without CFZ (1 μM) for 3 additional days. Levels of Htt-Q_94_ (measured by the CFP signal), TUBB3 (yellow) and MitoTracker (red) are shown. Hoechst (blue) was used to stain DNA and detect nuclei. An image of the entire well for this dataset, as well as the quantification of cell numbers is shown in Fig. S3. Scale bar (white), represents 15 μm. (**b**) HTM-dependent quantification of the cytoplasmic MitoTracker signal per cell from the experiment defined in (**a**). Error bars indicate SD and dashed lines indicate median values (n = 3). ∗*p* < 0.05, ∗∗∗*p* < 0.001, t-test. (**c**) Percentage of transdifferentiated neurons obtained from adult human dermal fibroblasts through direct reprogramming, in the presence or absence of the indicated doses of CFZ. Each dot represents the average value for an individual donor cell line (n = 7 CTRL lines, 84 wells analyzed in total). (**d**) Normalized neurite counts (% of control) in induced neurons treated with CFZ (n = 7 CTRL lines, 84 wells analyzed in total). (**e**) Normalized Mitotracker signal (% of control) in the neurites of induced neurons, in the presence of CFZ. Each dot represents the average values of individual donors. (n = 7 CTRL lines, 42 wells were analyzed in total) ∗∗*p* < 0.01, One-way ANOVA. All data are shown as mean ± SEM.

To further document the effect of CFZ in a neuronal model, we first used SH-SY5Y neuroblastoma cells, which can differentiate into a neuron-like phenotype with retinoic acid (RA).^67^ Parental cells were infected with pLVX-UbC-rtTA-Htt-Q94-CFP lentiviruses, enabling dox-dependent expression of Htt-Q_94_-CFP (SH-SY5Y^Q94^). After 5 days of differentiation with 10 μM RA, SH-SY5Y^Q94^ cells were treated with dox for 3 additional days. As in all previous models, Htt-Q_94_ expression had a profound impact on SH-SY5Y^Q94^ cells, as exemplified by decreased cell numbers and a reduction in the MitoTracker signal, all of which were rescued by treatment with CFZ (Fig. 5a and b and Fig. S4). Of note, CFZ had a significant effect in increasing cell numbers and mitochondrial activity also in SH-SY5Y^Q94^ cells that were not previously exposed to dox, although we cannot discard that this is due to small amounts of Htt-Q_94_-CFP being expressed even in the absence of dox.^68^ In any case, CFZ also increased the Mitotracker signal in induced neurons (iNs) generated by direct reprogramming of 7 adult human dermal fibroblasts, which better recapitulate human aging and disease phenotypes,^32^^,^^69^, ^70^, ^71^ without significantly affecting conversion rates or neuronal differentiation (as measured by the formation of neurites) (Fig. 5c–e and Fig. S5).

### Clofazimine rescues polyQ toxicity in worms and developing zebrafish

**Fig. 6:**
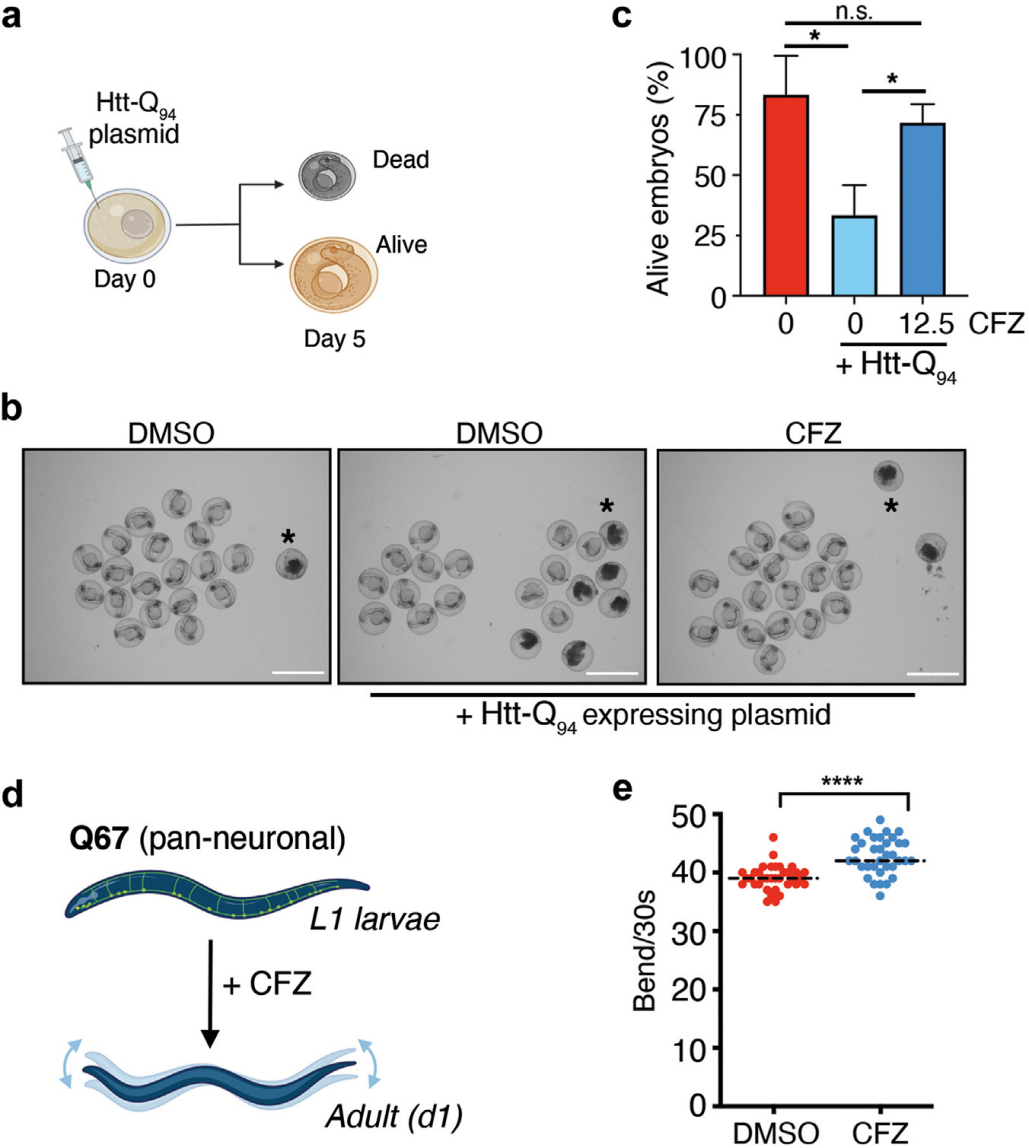
**CFZ rescues polyQ toxicity in worms and developing zebrafish.** (**a**) Scheme illustrating the pipeline followed to evaluate Htt-Q_94_ in developing zebrafish. Viability was monitored 5 days after microinjection with an Htt-Q_94_-CFP expressing plasmid. (**b**) Representative images of zebrafish embryos 24 h after microinjection of the Htt-Q_94_-CFP expressing plasmid or DMSO. Note the accumulation of dead embryos (black asterisk) upon Htt-Q_94_-CFP expression, which was significantly rescued by CFZ (12.5 μM). Scalebar (white) represents 1 mm. (**c**) Quantification from the experiment defined in (**a,b**). Error bars indicate SC (n = 3). ∗*p* < 0.05, ∗∗*p* < 0.01, t-test. (**d**) Scheme illustrating the pipeline used to evaluate the effect of CFZ in a worm model of polyQ toxicity. L1 larvae Q97 worms, presenting pan-neuronal expression of Q67-YFP, were grown in the presence of DMSO or 5 μM CFZ. When animals reached the adult stage (1d), their motility was quantified by measuring the number of bends per 30 s. (**e**) Quantification of data from (**d**). The experiment was done in triplicate, and a representative one is shown. Dashed lines indicate median values. ∗∗∗∗*p* < 0.0001, t-test.

Next, we tested if clofazimine could rescue the toxic effects of polyQ peptides in vivo. To this end, we first tested the effect of CFZ on polyQ toxicity in zebrafish. To this end, we used a previously developed plasmid enabling the expression of Htt-Q_94_-CFP.^34^ On day 0, fertilized zebrafish eggs were injected with the plasmid and exposed to CFZ at 12.5 μM (Fig. 6a). Consistent with previous studies,^72^^,^^73^ transgenic Htt-Q_94_ expression led to substantial embryonic lethality in developing zebrafish. Importantly, CFZ significantly increased the survival of Htt-Q_94_-transgenic embryos, confirming its ability to alleviate polyQ toxicity in vivo (Fig. 6b and c).

Finally, to assess whether CFZ could ameliorate polyQ toxicity in an animal model of the disease, we tested its effects in a *Caenorhabditis elegans* (*C. elegans*) model expressing expanded polyQ in neurons. Specifically, we used a strain expressing Q67-YFP fusion under the neuronal-specific RasGRP promoter (rmIs284[pF25B3.3::Q67::YFP] strain; Q67 hereafter) (Fig. 6d). Importantly, exposing the animals to CFZ from development significantly improved the motility defects previously reported in adult Q67 worms^52^ (Fig. 6e).

## Discussion

As mentioned in the introduction, and despite the substantial advances made in understanding the molecular basis of polyQ-diseases, this has not yet led to effective treatments. Among others, substantial efforts are being dedicated to finding therapeutic strategies to either reduce the expression of polyQ-containing proteins (e.g., antisense oligonucleotides (ASOs) or RNA interference), or that aim to either prevent the formation of polyQ aggregates or promote their clearance (reviewed in^29^). Our approach was rather to identify molecules capable of reducing the toxicity of polyQ-bearing proteins. In this regard, a similar approach was conducted by the Taylor laboratory, who searched for molecules that reduced apoptosis triggered by the expression of a truncated androgen receptor containing a 112-glutamine repeat in HEK 293T cells.^74^ In our screening model, U2OS and Htt-Q_94_ expression did not trigger apoptosis, but rather cell cycle arrest. Interestingly, we observed that the severity of this phenotype was more acute when cells were seeded at low densities, perhaps reflecting that the formation of polyQ aggregates is also enhanced at sub-confluence.^75^

The usefulness of our screening approach is supported by the fact that we identified compounds previously known to modulate the severity of polyQ pathology in preclinical models such as TZD.^26^^,^^56^^,^^57^^,^^59^ Unfortunately, TZD was later removed from the market because of hepatic toxicity, despite being originally approved for the treatment of diabetes.^76^ In this regard, we should note that the beneficial effects we observe for CFZ are also dose-dependent, as the compound also shows toxicity above a certain dose (which varies, depending on the assay). Nevertheless, cumulative data supporting that activation of the PPARγ/PGC1α axis is a potentially effective therapeutic approach for the treatment of neurodegenerative diseases^68^ emphasizes the need to discover additional PPARγ agonists that hopefully overcome the initial toxicities. In this regard, our work indicates that CFZ is a PPARγ agonist, with a similar binding affinity as TZD, but which is seemingly safe as it is in clinical use for the treatment of infectious diseases. Of note, one limitation of CFZ is its poor efficacy in crossing the blood–brain barrier (BBB), which has limited its use for the treatment of infections in the central nervous system. In this regard, there are already efforts dedicated to circumventing this problem such as nanoparticle-based formulations of CFZ.^77^ Nevertheless, our work already indicates that CFZ could be a useful alternative to TZD for the treatment of pathologies outside the CNS. In summary, our study further indicates the potential of PPARγ stimulation to reduce the severity of pathologies of polyQ-diseases, and that these effects are primarily related to mitochondrial function. In addition, our work provides an example of the possibilities offered by drug repurposing to identify medically approved drugs that could be investigated in the context of neurodegenerative diseases. While acknowledging the current pharmacological limitations of this drug, we believe that exploring the efficacy of CFZ or its derivatives in polyQ diseases deserves further preclinical work.

## Contributors

X.L. contributed to most experiments and data analyses and to the preparation of the figures. I.H. and J.J.L. helped with animal models. S.K. and D.V. performed the experiments and analyses in *C. elegans*. K.B., A.A.A., S.B., A.G. and K.P. contributed to the direct reprogramming experiments. M.H. helped with the chemical screen. L.L. provided technical help in several of the in vitro experiments. L.L. helped with zebrafish experiments. J.C.-P. contributed to the original design of the screen. D.H. contributed to most of the analyses and supervision of the project. O.F-C. supervised the study and wrote the MS. All authors reviewed and approved the final version of the manuscript. The corresponding author attests that all listed authors meet authorship criteria and that no others meeting the criteria have been omitted. O.F.-C. and D.H. have verified the underlying data.

## Data sharing statement

RNA sequencing data associated to this work are accessible at the NCBI Gene Expression Omnibus repository, under accession number GSE222758.

## Declaration of interests

The authors declare no competing interests.

## Appendix A Supplementary data

Supplementary material

POLYQ_Li
