# Supplementary figures and images for: The anti-leprosy drug clofazimine reduces polyQ toxicity through activation of PPARγ
Source: eBioMedicine. 2024 May 2;103:105124. doi: 10.1016/j.ebiom.2024.105124 (PMC11088276; doi:10.1016/j.ebiom.2024.105124)

**a** Raw data for Fig. 2c

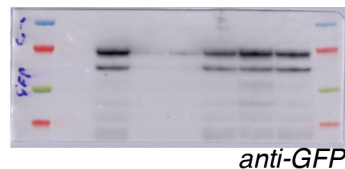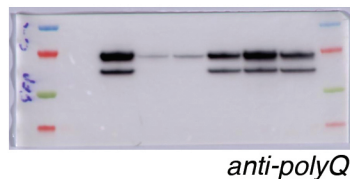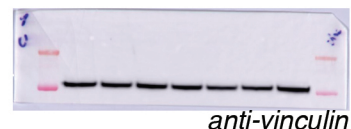

**b** Raw data for Fig. 4d

DMSO

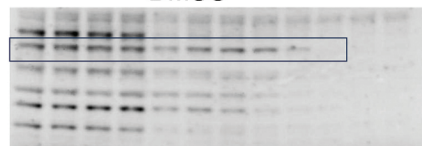

CFZ

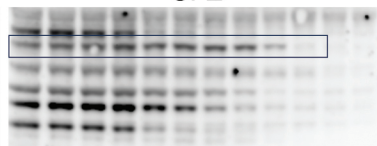

TZD

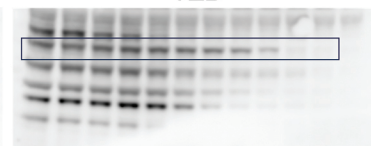

**c** Raw data for Fig. S2b

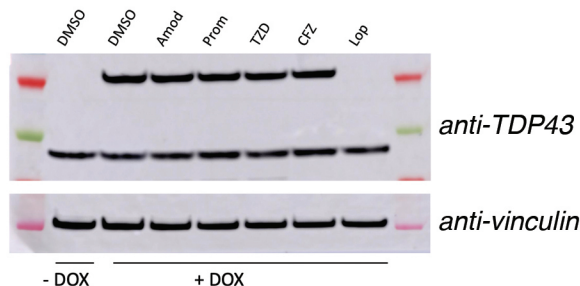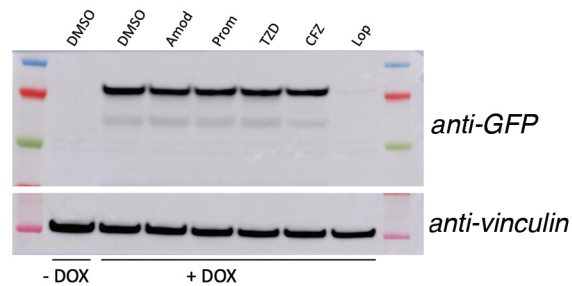

Supplement: Supplementary material [file mmc1.pdf]
