# Supplementary material for: The anti-leprosy drug clofazimine reduces polyQ toxicity through activation of PPARγ
Source: eBioMedicine. 2024 May 2;103:105124. doi: 10.1016/j.ebiom.2024.105124 (PMC11088276; doi:10.1016/j.ebiom.2024.105124)
Supplement: POLYQ_Li [file mmc2.pdf]

Li *et al.* **Fig S1**

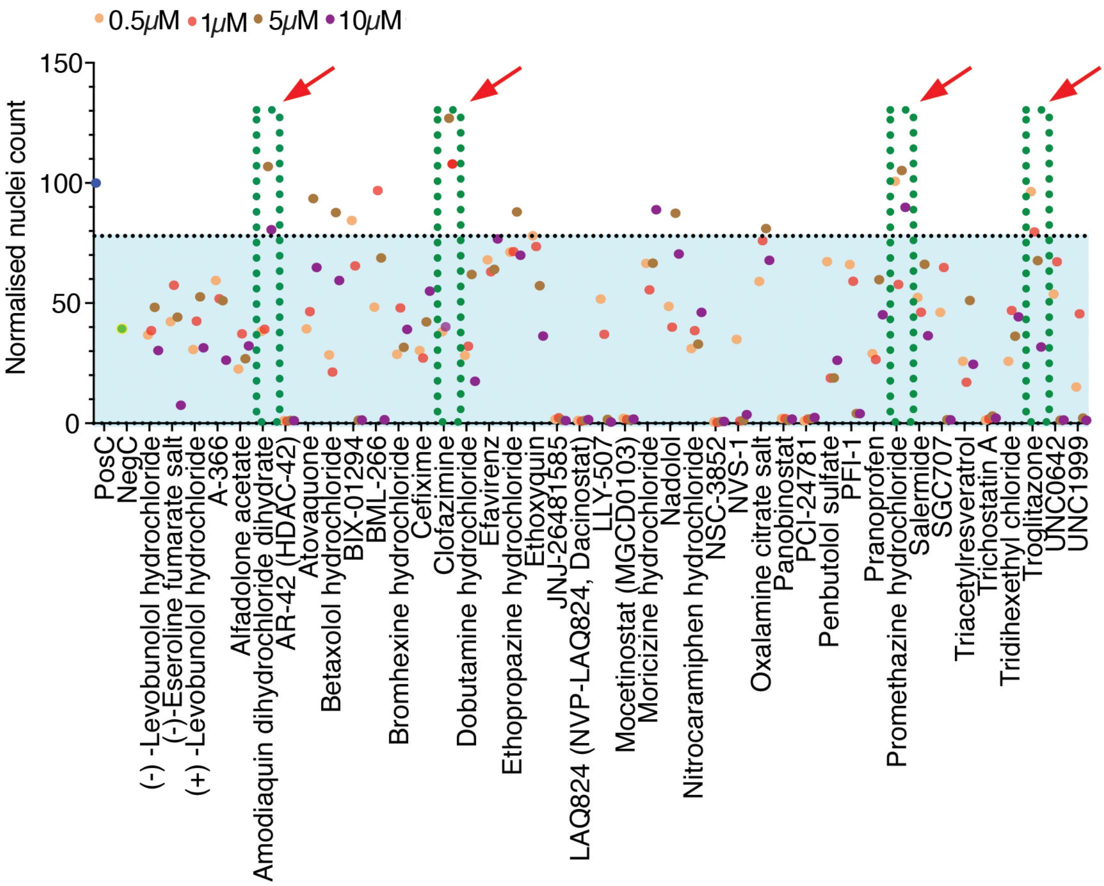

Figure S1. **Dose-response evaluation of the primary hits.** 35 hits identified in our primary screen (described in **Fig. 1**) as potentially increasing nuclei numbers in dox-treated U2OS^{Q94} cells, were evaluated using the same pipeline at increasing doses (0, 0.5, 1, 5 and 10 μM). Nuclei numbers were quantified at day 8 by HTM. Data show that four of the initial hits (highlighted by a red arrow) presented a dose-response increase in nuclei numbers in dox-treated U2OS^{Q94} cells, and were selected for further analyses.

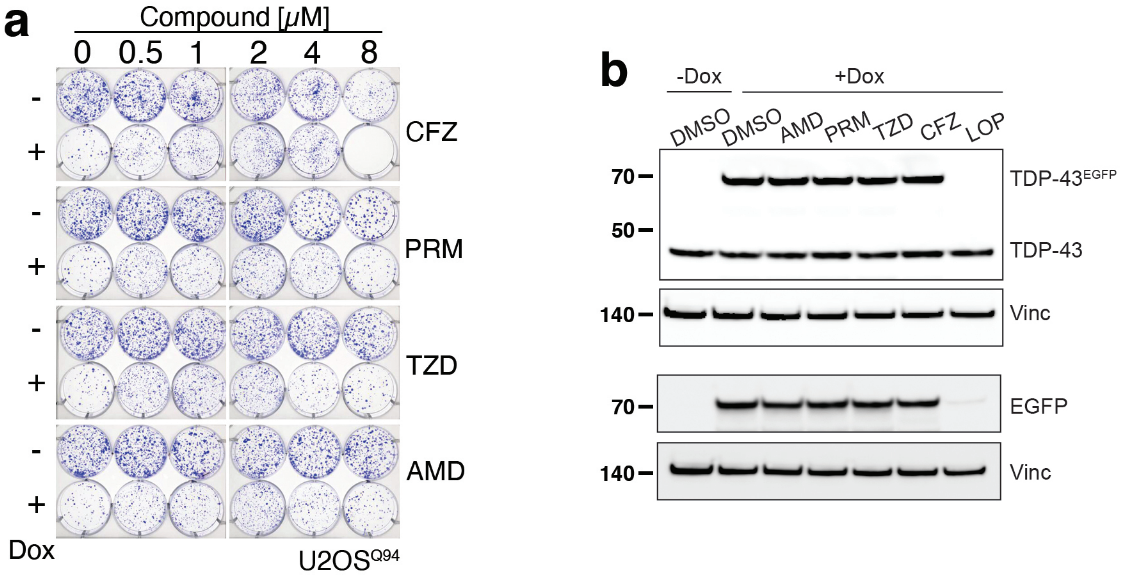
a
Compound [μM]
0
0.5
1
2
4
8
-
+
CFZ
PRM
TZD
AMD
Dox
U2OS Q94
b
-Dox
+Dox
DMSO
DMSO
AMD
PRM
TZD
CFZ
LOP
70
50
140
70
140
TDP-43 EGFP
TDP-43
Vinc
EGFP
Vinc

Figure S2. **Clonogenic assays of the 4 initial hits.** (**a**) Images from the full dose-response clonogenic survival assays performed in U2OS^{Q94} cells, treated or not with dox (50ng/ml) and the indicated drugs for 12 days. (**b**) WB analyses of the effect of the indicated compounds (AMD, PRM and TZD at 1μM, CFZ at 3μM), 2 days after inducing TDP-43EGFP expression with dox in an inducible clone of U2OS cells. Loperamide (10 μM) was used as a control, as we previously shown it inhibits dox-induced gene expression.

Li *et al.* **Fig S3**

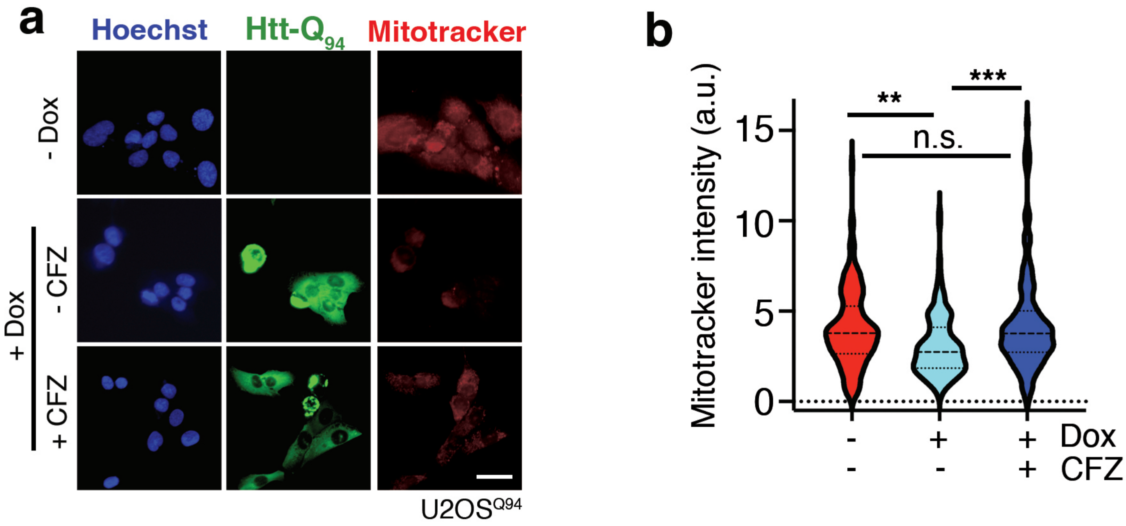

Figure S3. **CFZ rescues mitochondrial mass in polyQ expressing cells.** (**a**) Representative images of Htt-Q_{94} expression (detected by the EGFP signal, green) or the mitotracker signal (red) in the of $U2OS^{Q94}$ cells treated or not with dox (50 ng/ml) and CFZ (5 μM). Nuclei were stained with Hoechst (blue). (**b**) HTM-dependent quantification of the cytoplasmic mitotracker signal per cell from the experiment defined in (**a**). (n=3) $^{**}p<0.01$, $^{***}p<0.001$, 1-way ANOVA.

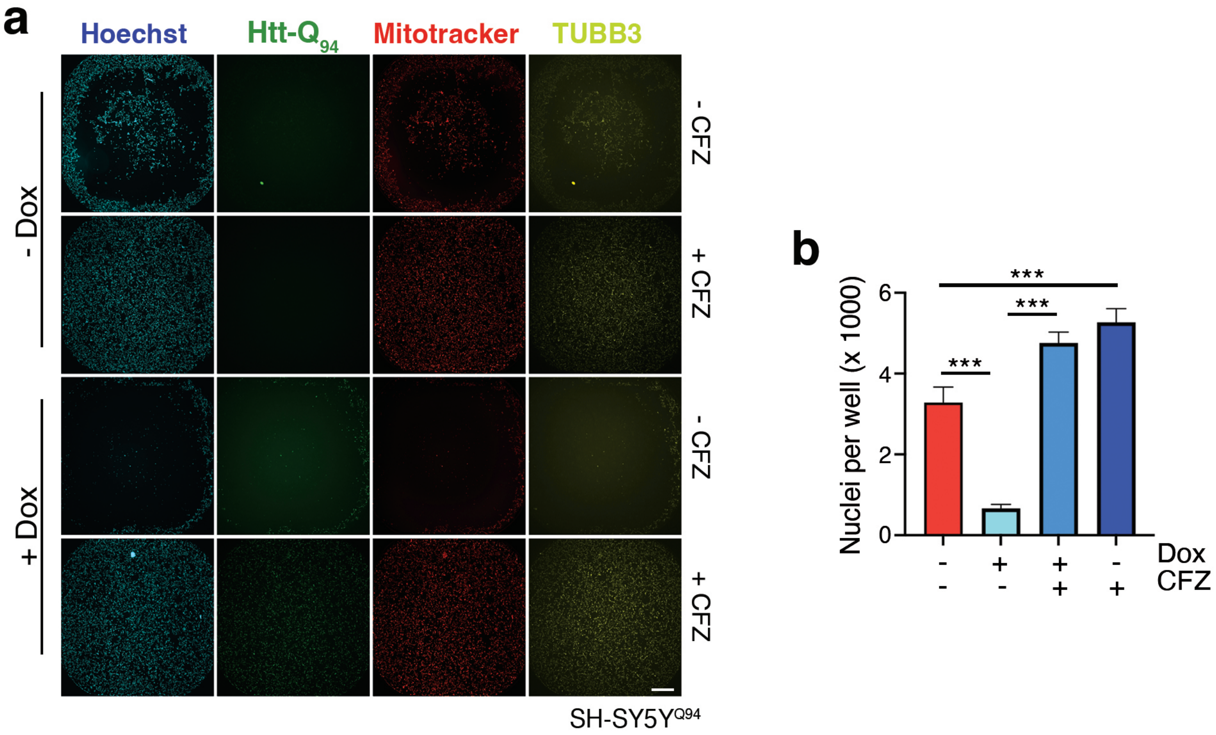
a
Hoechst
Htt-Q94
Mitotracker
TUBB3
- Dox
+ Dox
- CFZ
+ CFZ
- CFZ
+ CFZ
SH-SY5YQ94
b
Nuclei per well (x 1000)
0
2
4
6
***
***
***
Dox
CFZ
- + + -
- - + +

Figure S4. **Rescue of polyQ toxicity by CFZ in SH-SY5Y^{Q94} cells.** (**a**) Representative images from the entire well of a 384-well plate of SH-SY5Y^{Q94} cells differentiated with RA (10 μM, 5 days), and subsequently treated with dox (35 ng/ml) with or without CFZ (1μM) for 3 additional days. Levels of Htt-Q_{94} (measured by the CFP signal), TUBB3 (yellow) and mitotracker (red) are shown. Hoechst was used to stain DNA and visualize nuclei. Magnified insets from this dataset are shown in **Fig. 5A**. (**b**) HTM-dependent quantification of the number of nuclei per well from the experiment defined in (**a**). Error bars indicate SD (n=3). ***p<0.001, n.s., t-test.

Li *et al.* **Fig S5**

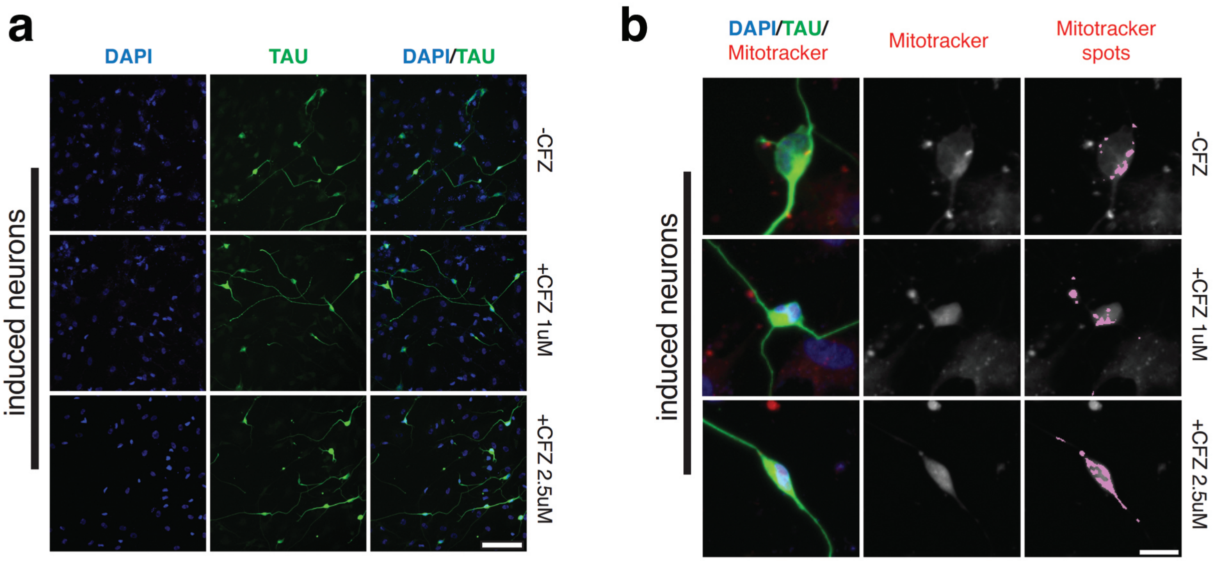

Figure S5. **CFZ increases MitoTracker intensity in human induced neurons.** (**a**) Induced neurons from 7 CTRL donors express the mature neuronal marker TAU independent of CFZ treatment. Scale bar = 100 µm. (**b**) Representative images illustrating a clear increase in the intensity of MitoTracker dots after 2.5 μM CFZ treatment. Scale bar = 20 µm.
